# Radiation-Induced Temporal Lobe Injury for Nasopharyngeal Carcinoma: A Comparison of Intensity-Modulated Radiotherapy and Conventional Two-Dimensional Radiotherapy

**DOI:** 10.1371/journal.pone.0067488

**Published:** 2013-07-10

**Authors:** Guan-Qun Zhou, Xiao-Li Yu, Mo Chen, Rui Guo, Ying Lei, Ying Sun, Yan-Ping Mao, Li-Zhi Liu, Li Li, Ai-Hua Lin, Jun Ma

**Affiliations:** 1 State Key Laboratory of Oncology in Southern China, Department of Radiation Oncology, Cancer Center, Sun Yat-sen University, Guangzhou, People's Republic of China; 2 State Key Laboratory of Oncology in Southern China, Imaging Diagnosis and Interventional Center, Cancer Center, Sun Yat-sen University, Guangzhou, People's Republic of China; 3 Department of Medical Statistics and Epidemiology, School of Public Health, Sun Yat-sen University, Guangzhou, People's Republic of China; West Virginia University School of Medicine, United States of America

## Abstract

**Background:**

To compare the radiation-induced temporal lobe injury (TLI) in patients with nasopharyngeal carcinoma (NPC) treated with intensity-modulated radiotherapy (IMRT) or two-dimensional conventional radiotherapy (2D-CRT).

**Patients and Methods:**

1276 cases of NPC treated with IMRT or 2D-CRT were retrospectively reviewed. A diagnosis of TLI was made on follow-up magnetic resonance imaging (MRI).

**Results:**

The crude incidence of TLI was 7.5% and 10.8% (*P* = 0.048), and the actuarial 5-year incidence was 16% and 34.9% (*P*<0.001) for the IMRT and 2D-CRT groups, respectively. Multivariate analysis revealed both T stage (*P*<0.001) and radiation technique (*P*<0.001) as independent predictors. Patients with T1, T2 and T3 disease had a significantly higher risk when treated with 2D-CRT (*P* = 0.005, 0.016, <0.001, respectively). This trend was not evident for T4 patients (*P* = 0.680). The 2D-CRT group had a longer latency for the development of TLI (*P*<0.001). Those with T4 disease had a shorter median time to TLI (*P* = 0.006, 0.042, <0.001 when compared with T1, T2 and T3, respectively).

**Conclusions:**

IMRT is superior to 2DRT for the management of T1-T3 NPC in terms of sparing the temporal lobe. The high incidence of TLI in T4 disease needs to be addressed.

## Introduction

Nasopharyngeal carcinoma (NPC) is a malignancy of the nasopharyngeal epithelium. Though relatively rare in most parts of the world, it is endemic in southern China with incidence rates between 15 and 50 per 100,000 [1].

Radical radiotherapy (RT) is the primary treatment modality for non-disseminated NPC due to its anatomic location and radiosensitivity. For decades, conventional two-dimensional radiotherapy (2D-CRT) was used via laterally opposed fields. Overall, disease control using 2D-CRT has been satisfactory. However, high-dose irradiation to critical structures proximate to the nasopharynx is associated with a high probability of toxicity.

In the management of NPC, incidental irradiation of the temporal lobe with consequent long-term injury is one of the most feared complications after radical radiotherapy, as it is can be devastating for patients and is associated with severe impairment of quality of life. In the series by Lee et al., 39% of the patients presented with vague symptoms such as mild dizziness, memory impairment, or personality change. Only 31% of patients with temporal lobe necrosis presented with classical temporal lobe epilepsy and 14% with non specific neurologic findings such as headache, mental confusion, or general seizures [2]. On physical examination, only a small percentage of patients showed signs of raised intracranial pressure such as papilledema or 6th nerve palsy [3]. Neurocognitive deficitcan may occur in patients with TLI. The general intelligence was usually intact [4]. Protection of the temporal lobe is difficult, particularly in patients with T3-4 disease who may have extensive skull base invasion or cavernous sinus involvement. The reported rates of temporal lobe injury (TLI) range from 3% to 40% [5]–[8], and TLI accounts for approximately 65% of deaths from radiation-induced complications [9].

The introduction of intensity-modulated radiation therapy (IMRT) has greatly improved our ability to sculpt radiation dose more precisely [10], [11]. IMRT technique can reduce the volume of high dose areas in the temporal lobes and thereby reduce risk of toxicities, compared with 2D-CRT [11]. Although dosimetric sparing of most parts of the temporal lobes is feasible with IMRT, data on the degree of benefit compared with 2D-CRT is lacking. In addition, knowledge on which patients may benefit most from IMRT is unknown.

Therefore, the aim of this study was to compare magnetic resonance imaging (MRI) findings of TLI in those treated with radical IMRT and with 2D-CRT within the same period in our institution.

## Materials and Methods

### Patient characteristics

Between January 2003 and December 2006, a total of 1276 patients with newly diagnosed, biopsy-proven nonmetastatic NPC patients who presented to the Sun Yat-sen University Cancer Center (Guangzhou, China) were included in the study. Approval for retrospective analysis of the patient data was obtained from the ethics committee of Sun Yat-sen University Cancer Center. Written consent was waived, while oral consent from the patients was obtained via telephone and documented by telephone recording. The use of oral consent was approved by the Institutional Review Board. Of the 1276 patients, 23 were excluded in order to ensure minimal heterogeneity in host factors and treatment modalities for the assessment of radiation-induced TLI. Excluded patients consisted of 16 with evidence of tumor directly invading the temporal lobe(s) and seven with adenocarcinoma. The study group therefore contained a total of 1253 patients. The male: female ratio was 2.9∶1 (943 men and 310 women), and the median age was 45 years (range, 11–78 years). Histological examination revealed that 99.4% of patients had World Health Organization (WHO) type 2 disease, 0.6% had WHO type 1 disease.

All patients underwent a pre-treatment evaluation that included a complete medical history, physical examination, hematology and biochemistry profiles, MRI of the neck and nasopharynx, chest radiography, abdominal ultrasonography, and a whole body bone scan using single photon emission computed tomography (SPECT). Positron emission tomography (PET)/CT was performed in 136 patients (10.9%). All patients were restaged according to the 2009 7^th^ UICC/AJCC staging system [12].

### Treatment

#### 2D-CRT

Details of the 2D-CRT techniques applied at our Cancer Center have been previously reported [13]. Patients were immobilized in the supine position with a thermoplastic mask and treated with two lateral opposing faciocervical portals to irradiate the nasopharynx and the upper neck in one volume, followed by application of the shrinking-field technique to limit irradiation of the spinal cord. An anterior cervical field was used to treat the neck with a laryngeal block. The accumulated radiation doses were 68 to 76 Gy (median, 70 Gy), with 2 Gy per fraction applied to the primary tumor, 60 to 64 Gy applied to involved areas of the neck, and 50 Gy applied to uninvolved areas. All patients were treated with one fraction daily, 5 days per week.

#### IMRT

The IMRT technique has been previously described [14]. In summary, the radiation dose prescribed in our institutional protocol is as follows: 68 Gy/30 fractions/6 weeks to the planning target volume (PTV) of gross tumor volume of the primary (GTV-P), 60 to 64 Gy to the PTV of nodal gross tumor volume (GTV-N), 60 Gy to the PTV of CTV-1 (high-risk regions), 54 Gy to PTV of CTV-2 (low-risk regions), and CTV-N (neck nodal regions). MRI was used to assist in defining the parapharyngeal and superior extent of the tumor. The treatment plan was optimized by Corvus version 3.0, an inverse IMRT planning system (Peacock, NOMOS Corp., Deer Park, IL). The treatment was delivered by a dynamic, multileaf, intensity-modulating collimator called MIMiC (developed by NOMOS Corp., Sewickly, PA). Tumor volumes of the nasopharynx and the upper neck were treated by IMRT over the entire course of treatment. For the lower neck, an anterior cervical field was used. All patients were treated with one fraction daily, 5 days per week.

#### Chemotherapy

During the study period, institutional guidelines recommended no chemotherapy for patients with Stage I to IIA disease, concurrent chemoradiotherapy for stage IIB, and concurrent chemoradiotherapy +/− neoadjuvant/adjuvant chemotherapy for stages III to IVa-b, as defined by the 6th edition of the UICC/AJCC staging system for NPC [15]. Overall, 473 patients were treated with RT alone and 780 patients received chemotherapy. Of the 856 patients with stage III/IV disease, 685 (80.0%) patients received chemotherapy.

In cases of documented relapse or persistent disease, salvage treatments including intracavitary brachytherapy, surgery and chemotherapy, where appropriate.

### MR imaging technique and image assessment

Magnetic resonance imaging was performed using a 1.5-Tesla system (Signa CV/i, General Electric Healthcare, Chalfont St. Giles, United Kingdom). The area from the suprasellar cistern to the inferior margin of the sternal end of the clavicle was examined with a head-and-neck combined coil. T1-weighted fast spin-echo images in the axial, coronal, and sagittal planes (repetition time, 500–600 ms; echo time, 10–20 ms), and T2-weighted fast spin-echo images in the axial plane (repetition time, 4,000–6,000 ms; echo time, 95–110 ms) were obtained before injection of the contrast material. After intravenous injection of gadopentetate dimeglumine (Gd-DTPA) (Magnevist, Schering, Berlin, Germany) at a dose of 0.1 mmol/kg body weight, spin-echo T1-weighted axial and sagittal sequences and spin-echo T1-weighted fat-suppressed coronal sequences were performed sequentially.

**Figure 1:**
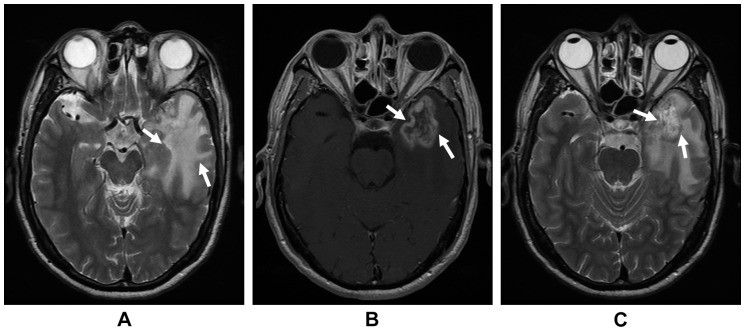
Representative MRI of patients with radiation-induced temporal lobe necrosis. (a) the T2-weighted axial image shows a finger-like lesion of increased signal intensity (between the arrows); (b) the post-contrast T1-weighted axial image shows a lesion with necrosis and heterogeneous signal abnormalities (between the arrows); (c) the T2-weighted axial image shows an oval cyst with high T2 signal intensity (between the arrows).

Two radiologists and a clinician specializing in head and neck cancers (Li L Ph.D., Liu LZ M.D. and Sun Y Ph.D.) separately reviewed the MR images. Any disagreements were resolved by consensus. A diagnosis of MRI-detected TLN required the following criteria: a) White matter lesions, defined as areas of finger-like lesions of increased signal intensity on T2-weighted images in the temporal lobe; b) Contrast-enhanced lesions, defined as lesions with or without necrosis on post-contrast T1-weighted images with heterogeneous signal abnormalities on T2-weighted images; and c) Cysts, round or oval well-defined lesions of very high signal intensity on T2-weighted images with a thin or imperceptible wall [16] (Figure 1).

### Follow up and statistical analysis

Patients were followed up at least every 3 months in the first three years and every 6 months thereafter. Routine follow-up care included complete head and neck examination, hematology and biochemistry profiles, chest radiography and abdominal sonography. Follow-up MRI of the nasopharynx and/or neck was performed every 6–12 months, especially when tumor recurrence was suspected or if complications of RT occurred. Interval times from the commencement of radiotherapy to the first detection of any components of TLI were calculated.

All analyses were performed using SPSS software, version 13.0 (SPSS, Chicago, IL). The pretreatment and treatment characteristics of the two groups were compared using the chi-square test. Both crude and actual rates of TLI were analyzed. The actuarial rates were calculated using the Kaplan-Meier method, and differences were compared using the log-rank test. Multivariate analyses with the Cox proportional hazards model were used to test the independent significance of different explanatory variables. Multiple comparisons were undertaken using the least significant difference t test (LSD-t test). A *P* value of 0.05 or less (in a two-sided test) was considered statistically significant.

## Results

Overall, there were 506 and 747 patients in the IMRT and 2D-CRT groups, respectively. The median follow-up time for survival analysis was 75.8 months, range 3.0 to 97.1 months. The 5-year overall survival rates were 83.2% and 76.7% for the IMRT and 2D-CRT groups, respectively, and the local recurrence-free rates were 92.7% and 86.8%, respectively.

### Incidence of TLN

The final follow-up MRI was performed on May 21, 2012. Available MR imaging findings revealed 38/506 and 81/747 cases of TLI in the IMRT and 2D-CRT groups, with crude incidence rates of 7.5% and 10.8%, respectively (*P* = 0.048).

**Figure 2:**
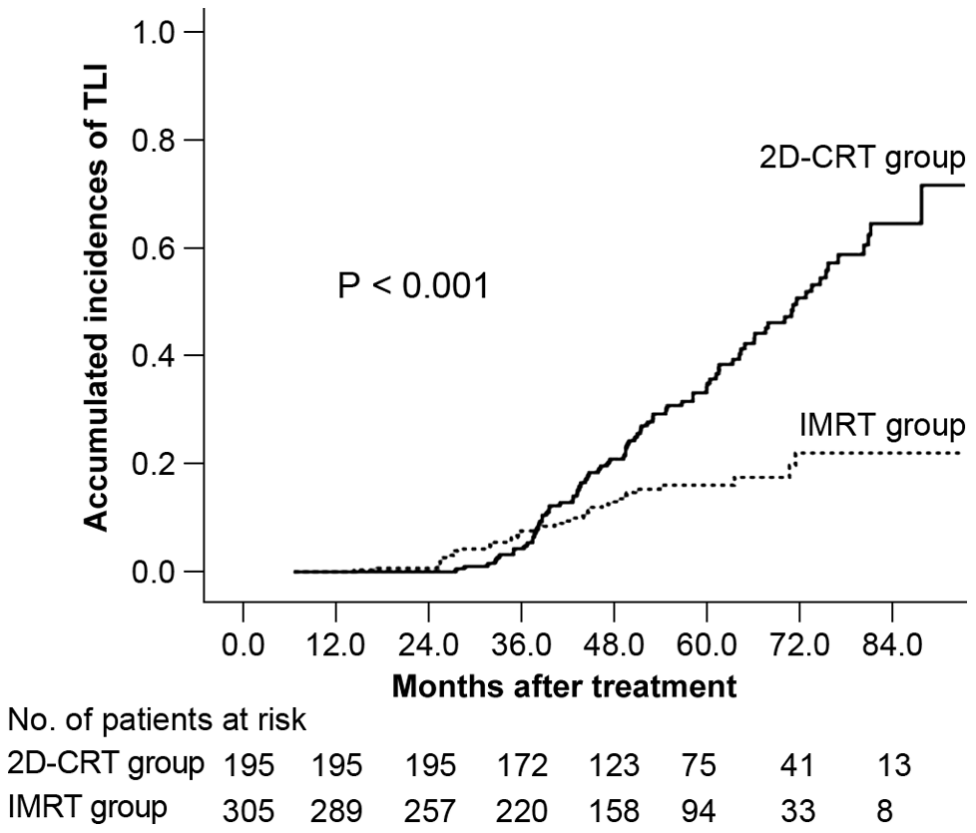
Kaplan-Meier curve of temporal lobe necrosis probability. Comparison of patients treated with intensity-modulated radiotherapy (IMRT) or conventional two-dimensional radiotherapy (2D-CRT).

Actuarial rates were analyzed based on patients who had completed at least 6 months follow-up post radiotherapy. Therefore, a total of 500 patients were eligible, with 305 and 195 in the IMRT and 2D-CRT groups, respectively. The median follow-up time for temporal lobe was 48.9 months (6.5 to 102.9 months) and 52.9 months (27.5 to 98.3 months) for IMRT and 2D-CRT group respectively. No significant differences in host factors, histological categories and tumor-related factors were identified between the two groups, apart from N (node) classification and chemotherapy (Table 1). The actuarial incidences of TLI were significantly different between the two groups, with rates at 1, 2, 3, 4, 5 and 6 years calculated as 0, 0.7%, 7.6%, 13.0%, 16.0% and 22.0% for the IMRT group, and 0, 0, 4.3%, 20.9%, 34.9% and 50.7% for the 2D-CRT group, respectively (HR [Hazard Ratio]  = 0.405, 95% CI for HR  =  [0.275, 0.596], *P*<0.001) (Figure 2).

**Table 1:** Characteristics of the study population treated by 2D-CRT and IMRT.

Characteristics	2D-CRT group (%) (*N* = 195)	IMRT group (%) (*N* = 305)	*P*
Age, y			0.065‡
<50	133(68.2%)	231 (75.7%)	
≥50	62(31.8%)	74 (24.3%)	
Gender			0.346‡
Male	141 (72.3%)	232(76.1%)	
Female	54 (27.7%)	73 (23.6%)	
Pathologic features			0.651‡
WHO Type 1	2 (1%)	2 (0.7%)	
WHO Type 2	193 (99%)	303 (99.3)	
T category*			0.241‡
T1	56 (28.7%)	157 (31.9%)	
T2	39 (20.0%)	74 (15.0%)	
T3	58 (29.7%)	123 (25.0%)	
T4	42 (21.5%)	138 (28.0%)	
N category*			0.082‡
N0	27 (13.8%)	71 (23.3%)	
N1	114 (58.8%)	158 (51.8%)	
N2	42 (21.5%)	59 (19.3%)	
N3	12 (6.2%)	17 (5.6%)	
Stage group*			0.019‡
I	10 (5.1%)	33 (10.8%)	
II	55 (28.2%)	79 (25.9%)	
III	81 (41.5%)	96 (31.5%)	
IVA-B	49 (25.1)	97 (31.8%)	
Chemotherapy			0.017‡
CRT	110 (43.6%)	203 (67.0%)	
RT alone	85 (56.4%)	100 (33.0%)	

Abbreviations: 2D-CRT  =  conventional two-dimensional radiotherapy; IMRT  =  intensity-modulated radiotherapy; WHO  =  World Health Organization; CRT  =  chemoradiotherapy; RT  =  radiotherapy.

* According to the American Joint Committee on Cancer, 7th edition.

‡ *P*-value calculated by the Chi-square test.

### Latency of TLI occurrence

**Figure 3:**
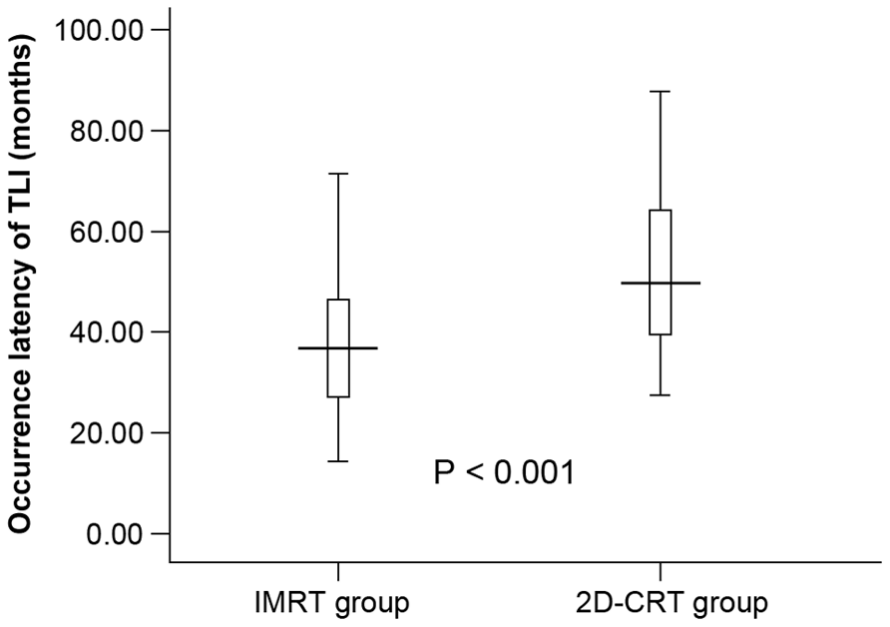
Latency of occurrence of temporal lobe necrosis. Patients treated with intensity-modulated radiotherapy (IMRT, left) vs. two-dimensional conventional radiotherapy (2D-CRT, right). Standard error bars are included and the thick horizontal line for each set of data points represents the median latent period.

The median time interval between the commencement of radiotherapy and the first MRI-detected TLI was 44.7 months (range, 14.4–87.6 months). The median latency of TLI detection in the IMRT group and in the 2D-CRT group was 36.85 and 49.77 months, respectively (*P*<0.001, Figure 3). The difference in latency for patients with different T classifications was statistically significant (*P*<0.001), and the median latency period for T1, T2, T3 and T4 was 49.4, 49.6, 54.2 and 39.5 months, respectively. Multiple comparisons between the groups revealed that T4 patients had a significantly shorter latency (*P* = 0.006, 0.042, <0.001 when compared with T1, T2 and T3, respectively, Table 2).

**Table 2:** Multiple comparison results of TLI latency between patients with different T classifications.

T classification		Mean difference	SE	*P* *	95% Confidences Interval	
					Lower	Upper
T1	T2	60.278	149.260	0.687	−235.38	355.93
	T3	−179.810	122.094	0.144	−421.66	62.03
	T4	319.278	114.268	0.006	92.94	545.62
T2	T3	−240.088	133.011	0.074	−23.38	503.56
	T4	259.00	125.865	0.042	9.69	508.31
T3	T4	499.088	92.035	0.000	316.79	681.39

* Calculated using the least significant difference t test (LSD-t test).

### Variables affecting the risk of TLN

Using univariate analysis of clinical characteristics and treatment-related parameters, four factors (radiotherapy technique, T classification, overall stage and chemotherapy) demonstrated a significant correlation with TLI (*P*<0.001, *P*<0.001, *P*<0.001 and *P* = 0.01, respectively). On the other hand, there was no association between age, gender, N classification, histology, hypertension, diabetes, and smoking and TLI (P = 0.586, 0.902, 0.136, 0.976, 0.553, 0.393 and 0.058, respectively).

Multivariate analysis was then performed to test independent significance, and the following parameters were included as covariables: radiation technique (IMRT vs. 2D-CRT), age (>50 years vs. ≤50 years), gender (male vs. female), histology (WHO type 1 vs. WHO type 2), T classification (T1-2 vs. T3-4), N classification (N0-1 vs. N2-3) and chemotherapy (yes vs. no). By forward inclusion of significant explanatory variables, both T classification (HR  = 2.777, 95% CI for HR  =  [1.841, 4.189], *P*<0.001) and radiation technique (HR  = 0.430, 95% CI for HR  =  [0.291, 0.635], *P*<0.001) were shown to be independent predictors of TLI.

### Subgroup analysis of the influence of radiation technique on different T classifications

**Figure 4:**
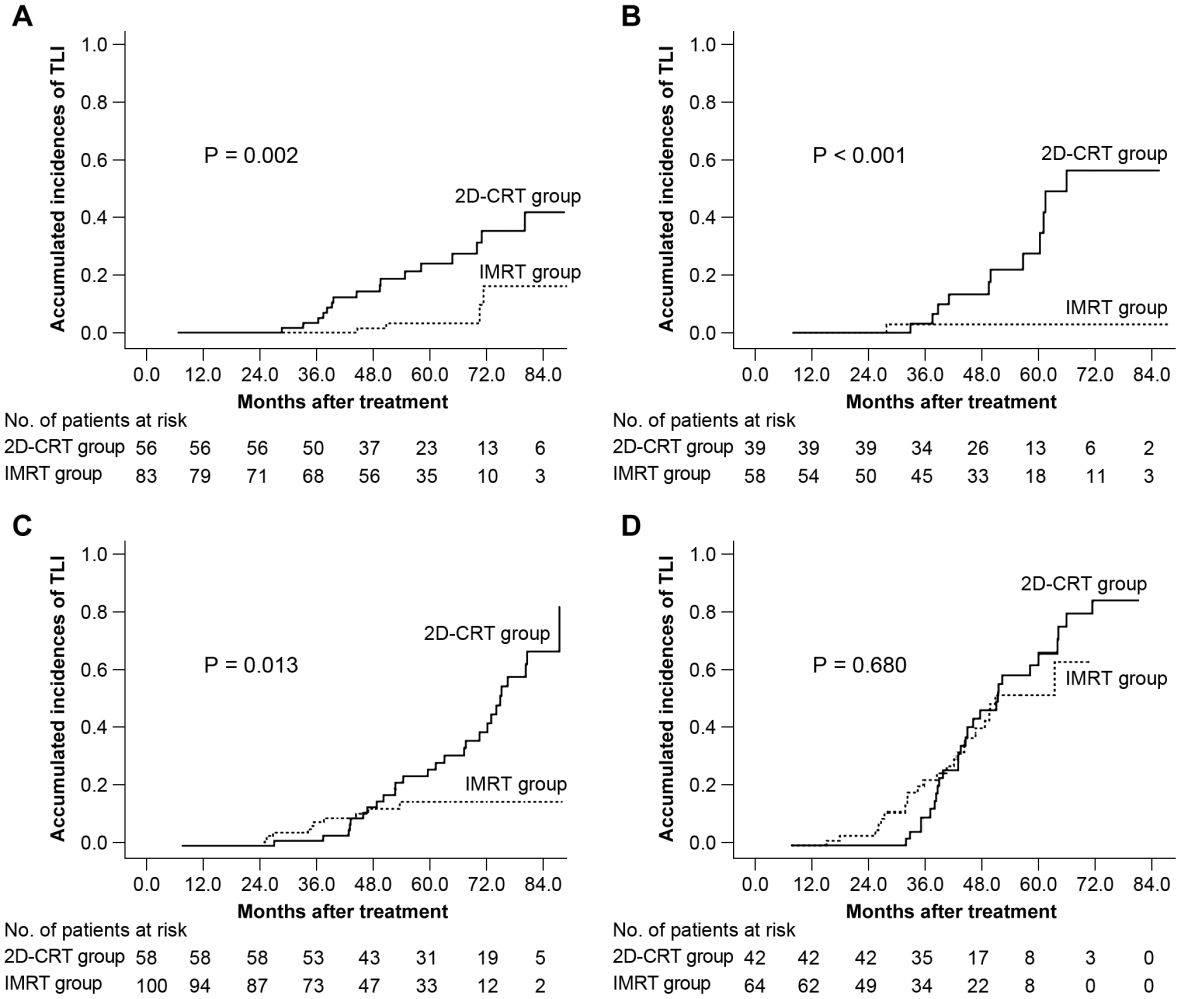
Kaplan-Meier curves comparing the probability of temporal lobe necrosis in T1-T4 patients. T1 disease (A), T2 disease (B), T3 disease (C), T4 disease (D) treated with intensity-modulated radiotherapy (IMRT) or two-dimensional conventional radiotherapy (2D-CRT).

The Kaplan-Meier method revealed a significantly higher risk of TLI for patients with T1, T2 and T3 disease when treated with 2D-CRT. In patients with T1 disease, the actual 5-year incidence of TLI in the IMRT group and in the 2D-CRT group was 1.6% and 24.5%, respectively (HR  = 0.174, 95% CI for HR  = [0.050, 0.607], *P* = 0.002) (Figure 4A). For T2 disease, the risk was calculated as 2.1% and 26.1%, respectively (HR  = 0.059, 95% CI for HR  =  [0.008, 0.449], *P*<0.001) (Figure 4B), and for T3 disease, 15.5% and 26.9%, respectively (HR  = 0.016, 95% CI for HR  =  [0.205, 0.848], *P* = 0.013) (Figure 4C). On the other hand, this trend was not evident in patients with T4 disease, with an incidence in the IMRT group and in the 2D-CRT group of 52.6% and 67.4%, respectively (HR  = 0.888, 95% CI for HR  =  [0.505, 1.562], *P* = 0.680) (Figure 4–D).

## Discussion

Today, IMRT is generally accepted as a more advanced radiation technique for the management of NPC. However, how effective IMRT is in sparing critical structures such as the temporal lobe is largely unknown. Comparison studies of the effects of IMRT and those of 2D-CRT on the reduction of the long-term complication, TLI, are lacking to date. Therefore, we conducted this retrospective study in order to directly compare IMRT and 2D-CRT in terms of radiation-induced TLI in patients treated in the same time period.

### The incidence of TLI

Fractional dose is generally considered to be highly significant in terms of TLI development, and our study presents a relatively higher incidence of TLI than has been reported in previous studies that used comparable fractional size [9], [17]–[18]. This inconsistency is probably due to the different diagnostic modalities utilized in different studies. The diagnosis of TLN typically lacks histological verification, and most studies have used subjective neurological symptoms as the major diagnostic criterion. However, the majority of affected patients tend to be either asymptomatic or to have very vague symptoms and no localizing signs, which leads to a misclassification bias and therefore an underestimation of the true incidence [8]. A diagnosis of TLI in our study was based on MRI, which was able to detect small necrotic nidus that may be missed when the diagnosis relies mainly on subjective neurological symptoms [19].

A further possible reason may be the method of calculating the incidence of TLI. To avoid the confounding effect of variability in the duration of follow-up, and the percentage of long-term survivors, most analyses in our study were based on actuarial incidence. Therefore, the cumulative incidence of TLI established in this study is more reliable than crude incidences alone [8].

### The benefit of IMRT on reducing radiation-induced TLI

Results from dosimetry studies have indicated that IMRT is capable of minimizing unnecessary doses to the temporal lobes [11]. IMRT significantly reduced the incidence of radiation-induced TLI compared with the 2D-CRT in our study. We hypothesize that the improvement observed with IMRT is largely due to the technical advantages of IMRT. Compared with opposed lateral fields in conventional 2D radiotherapy, modern IMRT utilizes multiple small segments of beams (pencil beams), and the intensities of the neighboring pencil beams vary. Collectively, beams composed of segments with different intensities produce dose distributions that conform to the required shape of the targets. Furthermore, the popular adopted simultaneous integrated boost (SIB) dosing regimen has the advantage of providing a more conformal dose distribution, thereby facilitating enhanced sparing of critical normal structures [20]. In addition, optimal target definitions in 3D planning of IMRT compared with 2D plans can by itself have an impact in reduction of high dose volumes in temporal lobes and thereby TLIs incidence.

### Room for improvement in the management of patients with T4 disease

IMRT significantly reduced the risk of TLI in patients with T1, T2 and T3 disease. On the other hand, the benefit of IMRT in patients with T4 disease was relatively limited. Of note is that our results were consistent with prospective data from Bakst et al [21], who reported that two of three patients who developed TLI after IMRT treatment had intracranial extension.

Appropriate radiation for patients with T4 disease and intracranial infiltration presents a difficult clinical challenge. Tight gradients between tumor targets and surrounding organs at risk (OARs) mean that a compromise between disease control and treatment-related side effects. Furthermore, the high-dose gradient of T4 tumors stresses the importance of setup accuracy during IMRT delivery [11], as even small setup errors can result in unacceptable radiation dose to nearby critical organs [22]. Given the proximity of the target areas to the temporal lobes in locally advanced NPC, the lobes are at risk of exposure to high doses and therefore susceptible to radiation-induced injury. The use of image guided radiation therapy (IGRT) and a safety margin around the OARs so called planning organ at risk volumes (PRVs) would be beneficial for the management of T4 disease [23].

### The latency of TLI

Our data demonstrated a median latency period of TLI occurrence of 36.85 and 49.77 months in the IMRT and 2D-CRT groups, respectively. These findings are consistent with previous studies that used similar radiation techniques. Reported median latency periods for patients treated with 2D-CRT using conventional fractions vary from 4.1 to 5 years [8], [9], while Bakst et al. [21] reported an even shorter latent period of 2 years in patients treated with radical IMRT.

This report suggests that patients treated with IMRT tend to develop TLI much earlier than those treated with 2D-CRT. Although the exact mechanisms associated with this observation remain unknown, a possible cause is the higher irradiation dose delivered to the temporal lobes in the IMRT group, as IMRT can be associated with compromised dosage uniformity and higher hot spots compared with conventional techniques [10]–[11]. As reported by Dörr [24], late radiation effects can occur after symptom-free latent periods lasting from months to many years, with an inverse dependence of latency on dose.

### Limitations

In order to avoid bias due to variability in the duration of follow-up, most statistical analyses were based on actuarial rates in this retrospective study. However, the time or frequency of MRI will undoubtedly influence the latency of TLI occurrence to some extent, as most patients with TLI had no or minimal symptoms and were detected only on follow-up MRI.

## Conclusion

To our knowledge, this is the first study to directly compare the influence of two different radiation techniques (IMRT and 2D-CRT) on the development of TLI. IMRT is currently the preferred technique of choice for the treatment of NPC, particularly considering the tissue sparing ability in terms of the temporal lobe. However, further improvements in the therapeutic ratio in T4 disease treated with IMRT need to be addressed, and the utilization of IGRT and appropriate PRVs may be helpful in this group of patients.

## References

[1] Jemal A, Bray F (2011) Center MM, Ferlay J, Ward E, et al (2011) Global cancer statistics. CA Cancer J Clin 61: 69–90. 21296855 10.3322/caac.20107

[2] Lee AW, Ng SH, Ho JH, Tse VK, Poon YF, et al (1988) Clinical diagnosis of late temporal lobe necrosis following radiation therapy for nasopharyngeal carcinoma. Cancer 61: 1535–42. 3349419 10.1002/1097-0142(19880415)61:8<1535::aid-cncr2820610809>3.0.co;2-e

[3] Lee AW, Sze WM, Fowler JF, Chappell R, Leung SF, et al (1999) Caution on the use of altered fractionation for nasopharyngeal carcinoma. Radiother Oncol 52(3): 207–11. 10580865 10.1016/s0167-8140(99)00116-4

[4] Cheung MC, Chan AS, Law SC, Chan JH, Tse VK (2003) Impact of radionecrosis on cognitive dysfunction in patients after radiotherapy for nasopharyngeal carcinoma. Cancer 97(8): 2019–26. 12673733 10.1002/cncr.11295

[5] Lee AW, Law SC, Ng SH, Chan DK, Poon YF, et al (1992) Retrospective analysis of nasopharyngeal carcinoma treated during 1976–1985: Late complications following megavoltage irradiation. Br J Radiol 65: 918–928. 1422667 10.1259/0007-1285-65-778-918

[6] Sham J, Choy D, Kwong PW, Cheng AC, Kwong DL, et al (1994) Radiotherapy for nasopharyngeal carcinoma: Shielding the pituitary may improve therapeutic ratio. Int J Radiat Oncol Biol Phys 29: 699–704. 8040015 10.1016/0360-3016(94)90556-8

[7] Lee AW, Foo W, Chappell R, Fowler JF, Sze WM, et al (1998) Effect of time, dose, and fractionation on temporal lobe necrosis following radiotherapy for nasopharyngeal carcinoma. Int J Radiat Oncol Biol Phys 40: 35–42. 9422555 10.1016/s0360-3016(97)00580-4

[8] Lee AW, Kwong DL, Leung SF, Tung SY, Sze WM, et al (2002) Factors affecting risk of symptomatic temporal lobe necrosis: significance of fractional dose and treatment time. Int J Radiat Oncol Biol Phys 53: 75–85. 12007944 10.1016/s0360-3016(02)02711-6

[9] Lee AW, Law SC, Ng SH, Chan DK, Poon YF, et al (1992) Retrospective analysis of nasopharyngeal carcinoma treated during 1976–1985: late complications following megavoltage irradiation. Br J Radiol 65: 918–928. 1422667 10.1259/0007-1285-65-778-918

[10] Xia P, Fu KK, Wong GW, Akazawa C, Verhey LJ (2000) Comparison of treatment plans involving intensity-modulated radiotherapy for nasopharyngeal carcinoma. Int J Radiat Oncol Biol Phys 48: 329–337. 10974445 10.1016/s0360-3016(00)00585-x

[11] Kam MK, Chau RM, Suen J, Choi PH, Teo PM (2003) Intensity-modulated radiotherapy in nasopharyngeal carcinoma: dosimetric advantage over conventional plans and feasibility of dose escalation. Int J Radiat Oncol Biol Phys 56: 145–157. 12694833 10.1016/s0360-3016(03)00075-0

[12] Edge SB, Byrd DR, Compton CC, Fritz AG, Greene FL, et al.. (2009) American Joint Committee on Cancer manual for staging of cancer [M]. 7th ed. Philadelphia: JB Lippincott.

[13] Lai SZ, Li WF, Chen L, Luo W, Chen YY, et al (2011) How does intensity-modulated radiotherapy versus conventional two-dimensional radiotherapy influence the treatment results in nasopharyngeal carcinoma patients? Int J Radiat Oncol Biol Phys 80(3): 661–8. 20643517 10.1016/j.ijrobp.2010.03.024

[14] Li WF, Sun Y, Chen M, Tang LL, Liu LZ, et al (2012) Locoregional extension patterns of nasopharyngeal carcinoma and suggestions for clinical target volume delineation. Chin J Cancer, 31 (12): 579–587. 10.5732/cjc.012.10095 PMC3777458 22854064

[15] American Joint Committee on Cancer (AJCC) (2002) AJCC Cancer Staging Manual, 6th edition. “Pharynx (including base of tongue, soft palate and uvula).” New York: Springer. 157–164.

[16] Wang YX, King AD, Zhou H, Leung SF, Abrigo J, et al (2010) Evolution of radiation-induced brain injury: MR imaging-based study. Radiology 254: 210–218. 20019142 10.1148/radiol.09090428

[17] Chatani M, Teshima T, Inoue T, Azuma I, Yoshimura H, et al (1986) Radiation therapy for nasopharyngeal carcinoma. Retrospective review of 105 patients based on a survey of Kansai Cancer Therapist Group. Cancer 57: 2267–2271. 3084058 10.1002/1097-0142(19860615)57:12<2267::aid-cncr2820571205>3.0.co;2-c

[18] Perez CA, Devineni VR, Marcial-Vega V, Marks JE, Simpson JR, et al (1992) Carcinoma of the nasopharynx: factors affecting prognosis. Int J Radiat Oncol Biol Phys 23: 271–280. 1587746 10.1016/0360-3016(92)90741-y

[19] Lee AW, Cheng LO, Ng SH, Tse VK, O SK, et al (1990) Magnetic resonance imaging in the clinical diagnosis of late temporal lobe necrosis following radiotherapy for nasopharyngeal carcinoma. Clin Radiol 42: 24–31. 2390834 10.1016/s0009-9260(05)81617-4

[20] Chen SW, Yang SN, Liang JA, Shiau AC, Lin FJ (2005) Comparative dosimetric study of two strategies of intensity-modulated radiother-apy in nasopharyngeal cancer. Med Dosim 30: 219–227. 16275564 10.1016/j.meddos.2005.07.001

[21] Bakst RL, Lee N, Pfister DG, Zelefsky MJ, Hunt MA, et al (2011) Hypofractionated dose-painting intensity modulated radiation therapy with chemotherapy for nasopharyngeal carcinoma: a prospective trial. Int J Radiat Oncol Biol Phys 80: 148–153. 20605352 10.1016/j.ijrobp.2010.01.026 PMC2952060

[22] Ballivy O, Parker W, Vuong T, Shenouda G, Patrocinio H (2006) Impact of geometric uncertainties on dose distribution during intensity modulated radiotherapy of head-and-neck cancer: the need for a planning target volume and a planning organ-at-risk volume. Curr Oncol 13: 108–115. 17576450 10.3390/curroncol13030010 PMC1891177

[23] Wang J, Bai S, Chen N, Xu F, Jiang X, et al (2009) The clinical feasibility and effect of online cone beam computer tomography-guided intensity-modulated radiotherapy for nasopharyngeal cancer. Radiother Oncol 90: 221–227. 18930327 10.1016/j.radonc.2008.08.017

[24] Dörr W (2010) Radiation effect in normal tissue – principles of damage and protection. Nuklearmedizin 49 Suppl 1S53–58. 21152682

